# DNA methylation drives hematopoietic stem cell aging phenotypes after proliferative stress

**DOI:** 10.1007/s11357-024-01360-4

**Published:** 2024-10-11

**Authors:** Hagai Yanai, Taylor McNeely, Saipriya Ayyar, Michael Leone, Le Zong, Bongsoo Park, Isabel Beerman

**Affiliations:** Epigenetics and Stem Cell Unit, Translational Gerontology Branch, National Institute On Aging, NIH, 251 Bayview Blvd, Suite 100/10C220, Baltimore, MD 21224 USA

**Keywords:** Hematopoietic stem cells, Aging, Replicative stress, DNA damage, DNA methylation

## Abstract

Aging of hematopoietic stem cells (HSCs) is implicated in various aging phenotypes, including immune dysfunction, anemia, and malignancies. The role of HSC proliferation in driving these aging phenotypes, particularly under stress conditions, remains unclear. Therefore, we induced forced replications of HSCs in vivo by a cyclical treatment with low-dose fluorouracil (5FU) and examined the impact on HSC aging. Our findings show that proliferative stress induces several aging phenotypes, including altered leukocyte counts, decreased lymphoid progenitors, accumulation of HSCs with high expression of Slamf1, and reduced reconstitution potential, without affecting stem cell self-renewal capacity. The divisional history of HSCs was imprinted in the DNA methylome, consistent with functional decline. Specifically, DNA methylation changes included global hypermethylation in non-coding regions and similar frequencies of hypo- and hyper-methylation at promoter regions, particularly affecting genes targeted by the PRC2 complex. Importantly, initial forced replication promoted DNA damage repair accumulated with age, but continuous proliferative stress led to the accumulation of double-strand breaks, independent of functional decline. Overall, our results suggest that HSC proliferation can drive some aging phenotypes primarily through epigenetic mechanisms, including DNA methylation changes.

**Supplementary Information:**

The online version contains supplementary material available at 10.1007/s11357-024-01360-4.

## Background

Hematopoietic stem cells (HSC) are responsible for producing all blood cell types throughout the lifespan. Many aging phenotypes have been described in HSCs including increased numbers, reduced functional potential, an altered epigenetic landscape, intrinsic myeloid bias, and others [1–4]. Recently, it has also been shown that the removal of the age-associated myeloid biased HSCs [5] results in a rejuvenation of several immune phenotypes [6]. However, it is still not fully understood what drives HSC aging. While it has been demonstrated that stem cell-extrinsic factors can play a part [7–10], several changes, such as genomic / epigenomic integrity, are most likely driven intrinsically. Among these potential aging drivers, several lines of evidence suggest that the replicative history of HSCs is a major factor. First, despite possessing an extremely high replicative potential [11–14], this ability appears to be finite [15]. In addition, HSCs are predominantly quiescent throughout life, especially during homeostasis absent of acute stress [16–19]. HSCs also seem to track and limit their self-renewal under normal physiological conditions, affecting their functional potential decline during aging [20]. Finally, experimentally enforcing HSC replication, by various approaches, leads to an altered epigenomic profile that mimics physiological aging [3], expansion of an age-associated sub-population [21], and when extreme, HSC attrition [22].

It has been previously shown that DNA damage accrues during aging in quiescent HSCs, which is mostly fixed by entry to the cell cycle [23]. However, others have reported that proliferation promotes DNA damage [22] or causes replicative stress that is independent of genomic integrity [24]. This yet unclear relationship between HSC replicative profile, genomic integrity, and aging is especially important in the context of human age-related diseases, as the evident age-related accrual of DNA damage [25] can result in somatic mutations in the progenitor compartment [26–29] that could contribute to clonal expansion and disease [30–33]. The same is true for clinical stem cell transplants especially since they would involve a heavy stem cell replication burden to fully reconstitute the recipient’s hematopoietic system.

Another cell-intrinsic feature that was proposed to contribute to HSC aging is DNA methylation changes [34]. Differential methylation has been reported in HSCs from old animals [3, 4, 35] and has been linked to HSC proliferation [3]. It has also been proposed that at least some aging DNA methylation changes are intrinsically driven rather than attributed to signaling from the niche [36], at least when predicting age with a DNA methylation aging clock. However, it is still unclear what drives the alteration of the methylome and which of the changes contribute to the functional decline of HSCs with age.

To better understand the relationship between proliferative stress and aging of HSCs, we used serial low-dose treatments of 5-Fluorouracil to force HSC proliferation in vivo. We analyzed the genomic integrity, gene expression, and DNA methylation profiles to better understand the mechanisms and dynamics of replicative stress.

## Methods

### Aim, design, and setting

The aim of this study was to better understand the effects of proliferation and proliferative stress on HSCs in an in vivo environment. Specifically, we wanted to measure the effects on HSC function in a native and transplant setting, DNA integrity, gene expression, and epigenetic regulation (DNA methylation).

### Animals

Unless otherwise described, all experiments used 3-month-old C57BL/6 J female mice (at study start), maintained at the NIA vivarium under standard conditions as depicted in the Guide for the Care and Use of Laboratory Animals. All experimental procedures were approved by the NIA (National Institute on Aging) Animal Care and Use Committee (469-TGB-2025).

5-FU was administered at 150 mg/kg by intraperitoneal injection, once per 3 weeks, as previously described [3]. Animals were continuously monitored for general health and body weight and no discernable side effects were observed.

### Hematological analysis

For hematological analysis, ~ 50 µl blood was drawn from the tail into an EDTA tube and immediately measured for complete blood counts using a Hemavet 950FS analyzer (Drew Scientific, FL, USA).

### Bone marrow analysis and HSC isolation

For bone marrow analysis, both hind legs, including the pelvis, femur, and tibia, were crushed with cold sample media (PBS, 2% FBS, 2 mM EDTA, Pen/Strep 100 µg/ml (ThermoFisher Scientific, USA)) then filtered with a nylon mesh. Marrow was then treated for red blood lysis by ACK treatment (30 s in RT) and stained with the following antibodies for 90 m in the dark on ice: CD34 (Thermo Fisher Scientific, 11–0341-85, 1/50), Flk2 (BioLegend, 135310, 1/50), cKit (BioLegend, 105808, 1/200), Sca-1 (BioLegend, 108126, 1/200), CD150 (BioLegend, 115914, 1/200), CD16/32 (Thermo Fisher Scientific, 45–0161-82, 1/100), IL-7Rα (BioLegend, 135024, 1/200) and biotin-conjugated lineage markers, including TER-119 (BioLegend, 116204, 1/100), B220 (BioLegend, 103204, 1/100), CD11b (BioLegend, 101204, 1/100), CD3 (BioLegend, 100244, 1/100), and Gr-1 (BioLegend, 108404, 1/100). Propidium Iodide (PI) was employed to exclude dead cells from the analysis, and HSCs were analyzed on a FACSAria II (BD Biosciences) flow cytometer.

For HSC isolation, c-Kit enrichment was first performed using c-Kit/PE antibody and EasySep™ PE immunomagnetic positive selection kit II (StemCell Technologies, 17684) according to manufacturer’s instructions. C-Kit enriched cells were then stained as described above only using antibodies against CD34, Flk2, c-Kit, Sca-1, CD150, IL-7Rα and lineage markers, including TER-119 (BioLegend, 116232, 1/200), B220 (BioLegend, 103227, 1/200), CD11b (BioLegend, 101224, 1/200), CD3 (BioLegend, 100214, 1/200), and Gr-1 (BioLegend, 108434, 1/200). PI was used for dead cell exclusion, and HSCs were sorted as PI-Lin^−^cKit^+^Sca1^+^CD34^−^Flk2^−^CD150^+^ on a FACSAria II (BD Biosciences) flow cytometer.

### HSC transplant

CD150^+^ HSCs were sorted as explained above from the 5FU treated CD45.2 donor mice and 400 HSCs were transplanted into lethally irradiated (954 rads), CD45.1 hosts (11–13 weeks old) along with 2 × 10^5^ CD45.1 competitor whole bone marrow cells. All transplants were performed by tail vein injection in a 200 µl PBS solution. Following transplantation, recipient mice were maintained on antibiotics (Trimethoprim-Sulfamethoxazole; TMS) in their drinking water for two weeks and monitored closely for health issues. Engraftment was assessed by peripheral blood chimerism from tail vein bleeds up to 4 m after transplant, at which point recipient mice were sacrificed for bone marrow analysis and secondary transplant. For post-transplant bone marrow analysis, CD45.1 (BioLegend, 110728, 1/100) and CD45.2 (BioLegend, 109820, 1/100) antibodies were included to differentiate donor- and host-derived cells.

### mRNA expression

For gene expression analysis, 25-50 K sorted HSCs were sorted into Trizol and kept frozen until RNA isolation. mRNA was assessed with the Affymetrix Clariom D platform at the Johns Hopkins Medical Institute Deep Sequencing and Microarray Core (MD, USA) under manufacturer standard operating procedures. Analysis was performed on the Transcriptome Analysis Console (TAC) software (Thermo Fisher Scientific, USA) using the standard Limma Affymetrix QC with SST-RMA normalization. Assessment of statistical dependence of expression as a function of treatment cycles was performed on the JMP statistical software (MP Statistical Discovery LLC).

### DNA damage analyses

For the alkaline comet assay, 5 K HSCs were sorted into PBS and the comet assay was performed according to manufacturer instructions (Trevigen – Bio-Techne, USA) and as previously described [3]. Comets were imaged on the DeltaVision scanning microscope (GE, USA). Images were then semi-manually quantified using CometScore 2.0 by 2 independent people in a double-blind fashion. Statistical assessment of group differences was performed on GraphPad Prism (Dotmatics, MA, USA).

gH2AX assay was performed as previously described [3] and imaged with the DeltaVision scanning microscope (GE, USA). Quantification was performed manually in a blind fashion using ImageJ and statistical analysis was performed as in the comet assay.

### DNA methylation analysis

For DNA methylation, HSCs were sorted into PBS and flash frozen until DNA methylation assay by reduced representation bisulfite sequencing as previously described [3]. Methylation data was analyzed as follows to identify differentially methylated loci: raw reads of adapter dimers were trimmed using Trim Galore (0.4.3) with a minimum quality score above > 25 then attached adapter dimers were trimmed using cutadapter. Bisulfite-converted index (GA and CT conversion) was generated using mm10 mouse genome and aligned with bismark [37]. Aligned reads were then analyzed with Bismark methylation extractor tool for the level of methylation in each CpG. At least a million sites per sample were predicted. Differentially methylated loci were predicted by methylKit [38] as at least > 10% methylation difference (FDR < 0.05) with a minimum coverage of ten reads per base.

To generate DNA methylation blocks, we generated 100 base-pair binned DNA methylation levels across the genome, calculated the average DNAm level per block for each experimental group (0x, 2x, 4x, and 10x) then used this average DNAm level for mean CpG imputation. We calculated the DNAm level using all methylated and unmethylated sites from the binned CpG sites when the minimum coverage was more than 10. Genome annotation was based on the Homer package. Genes, exons, introns, and UTRs were taken from Homer annotation tools. TSS-promoter sites were considered the identified DMRs close to TSS (less than 1 kb). TSS-proximal sites were considered the identified DMRs close to TSS (less than 20 kb), and TSS-distal sites were considered the DNAm block whose distance to TSS is above 20 kb. Correlation and DNAm heatmap plots were generated by ggplot2, heatmap.2 function in R environment. (R version 4.2.0). Response screening was performed in JMP. Upset plots were generated on Intervene (https://intervene.shinyapps.io/intervene/). Graphical representation of DNA methylation results was plotted on Biorender (https://www.biorender.com/). Enrichment analyses were performed using GSEA (https://www.gsea-msigdb.org/gsea/login.jsp).

## Results

### Induced HSC proliferation by serial 5-fluorouracil treatments mimics some hematopoietic and bone marrow aging phenotypes

**Fig. 1:**
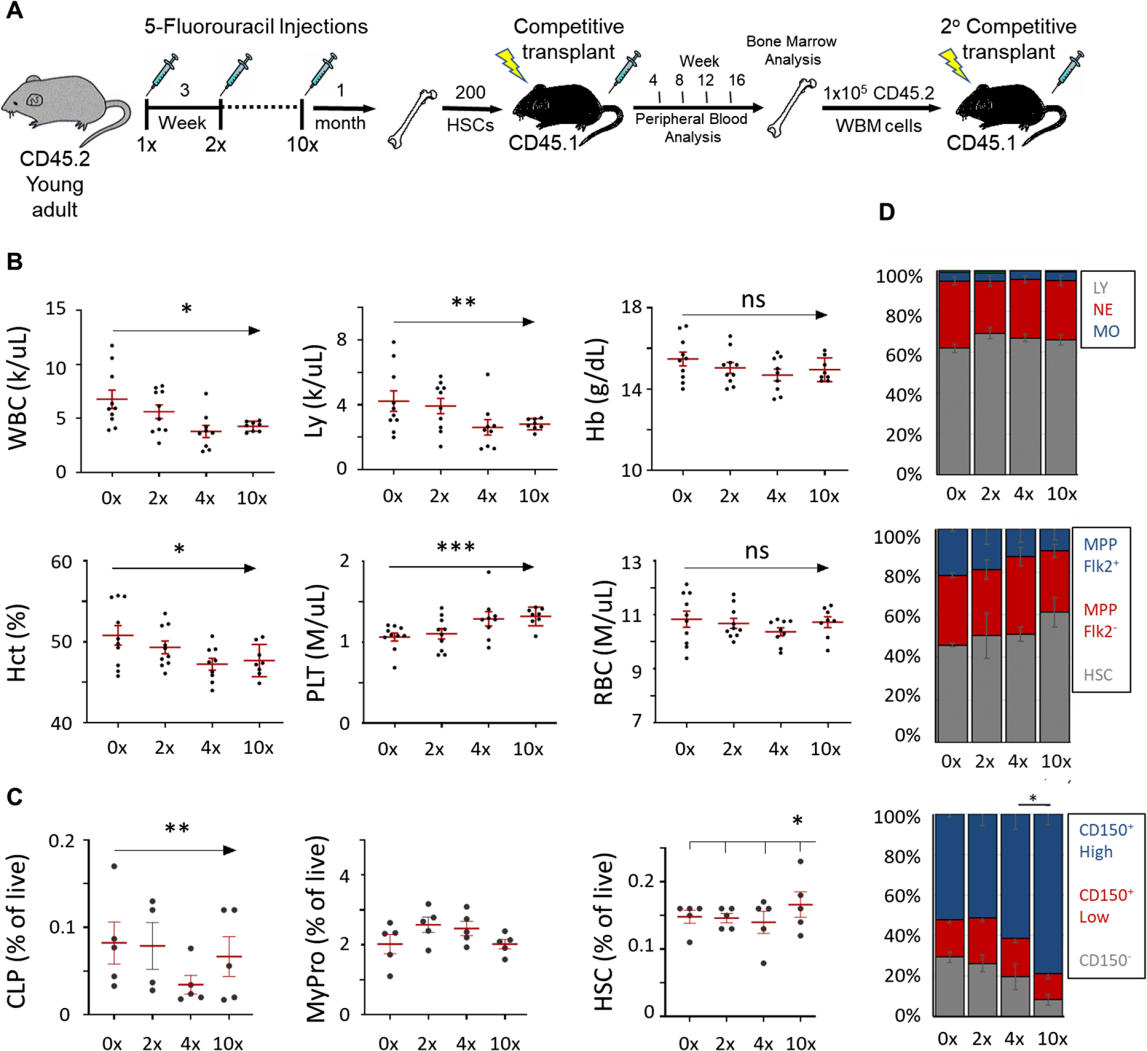
Enforced HSC replications lead to several hematopoietic aging phenotypes. **A** Study design. **B** Complete blood counts (CBC) 1 month after the end of treatment for white blood cells (WBC), lymphocytes (Ly), hemoglobin (Hb), hematocrit (Hct), platelets (PLT), and red blood cells (RBC). Each dot represents a single mouse with red lines representing mean ± SEM. **C** Bone marrow progenitor frequencies for common lymphoid progenitors (CLP), myeloid progenitors (MyPro), and hematopoietic stem cells (HSC). **D** Proportions of peripheral blood cells (top), major populations in the bone marrow multipotent compartment (LSK – middle), and HSC subpopulations based on CD150 expression (bottom). Statistical significance of treatment was determined by a one-way ANOVA (*p < 0.05; **p < 0.01; ***p < 0.001)

Adult HSCs are predominantly quiescent [16–19]. Therefore, to efficiently investigate the impact of in vivo replications on HSCs, we treated young adult mice with 150 mg/Kg of 5-fluorouracil (5-FU), which preferentially kills cycling blood cells and drives HSC proliferation, as previously described [23, 39]. To test for different degrees of proliferation, we treated mice in cycles of zero (0x) to ten (10x) serial doses, with 3-week intervals between injections to allow the hematopoietic system to regain homeostasis (Fig. 1A). 5-FU treatments had no effect on body weight (Fig. S1A) and mice had no overt side effects. To measure the long-term effects of proliferative stress on HSCs on hematopoiesis, we measured complete blood counts (CBC) 1 month following the last 5FU induction cycle. We observed a progressive, regimen-dependent reduction in white blood cells, lymphocytes, and hematocrit, and an increase in platelet counts (Fig. 1B), corresponding to the observed changes in the blood of 2-year-old mice (Fig. S1B). The overlap in aging-like changes in the peripheral blood after 5FU were not universal though, as red blood cell numbers, distribution width, and hemoglobin levels remained unaffected by the treatment yet change with age.

Aging of the bone marrow progenitor compartment includes a reduction in lymphoid progenitor frequencies and an accumulation of HSCs that express a high level of the surface marker Slamf1 (CD150) [5]. We observed a proliferative stress-dependent trend of common lymphoid progenitor reduction in the bone marrow, while maintaining myeloid progenitor frequencies (Fig. 1C, Fig. S2). Accordingly, the fraction of CD150^high^ HSCs was increased (Fig. 1D), but only a mild increase in overall HSC frequencies was observed after 10 cycles. This is likely because the age-related accumulation of CD150^high^ HSCs is an early aging phenotype that was already present in 0 × controls at the endpoint of the experiment when the animals reached early middle age (Fig. 1C, Fig. S3). This bone marrow phenotype was consistent with the myeloid bias in the peripheral blood of all experimental animals (Fig. 1D), corresponding to the aging phenotype (Fig. S3).

### HSC functional potential is decreased following forced proliferative stress in a transplant setting

**Fig. 2:**
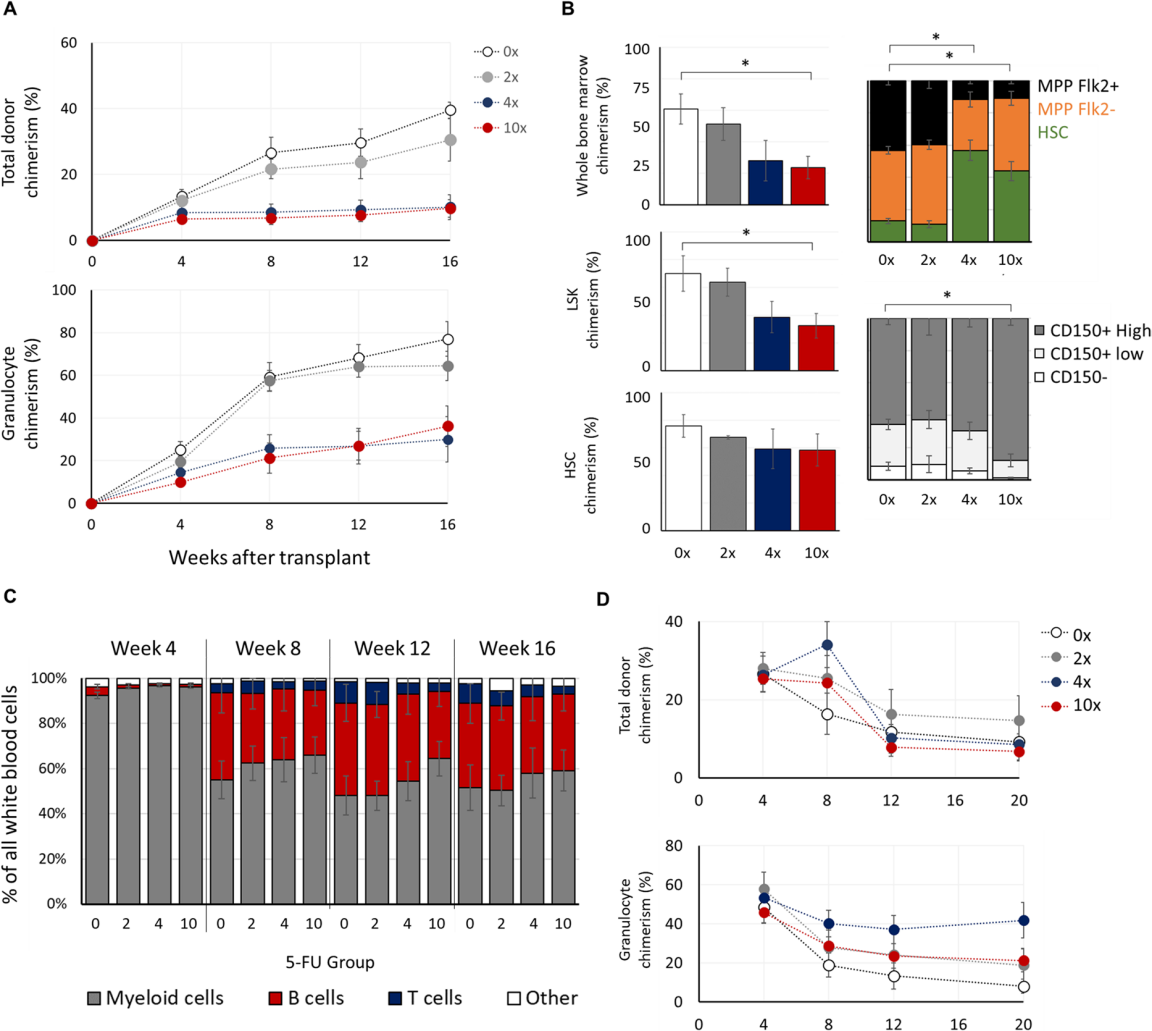
Enforced HSC replications lead reduced reconstitution potential but not self-renewal. HSCs from the indicated treatment groups were injected into lethally irradiated host CD45.1 mice (400 cells per host) with 200 K bone marrow cells from young CD45.1 donor as competitors. **A** Total blood chimerism and granulocyte-specific chimerism (determined as the ratio of CD45.2/CD45.1) at the indicated time points. Each dot represents group mean ± SEM. **B** Bone marrow progenitor chimerism for the indicated populations 4 months after transplant (left) and donor-derived population distributions for the multipotent progenitor compartment (top), and HSC compartment (bottom). **C** Peripheral blood population distribution for donor-derived cells. **D** Total and granulocyte chimerism following a secondary transplant

To test how forced proliferations influence the functional capacity of HSCs, we performed competitive transplants of stem cells into young lethally irradiated mice (Fig. 1A). HSCs from donors that received four or more serial treatments of 5-FU displayed a marked reduced reconstitution potential, as seen by both total blood and granulocyte chimerism (Fig. 2A). These HSCs also demonstrated a reduced capacity to reconstitute the bone marrow and the primitive multipotent compartment (Fig. 2B). However, all experimental groups had similar potency in reconstituting the HSC compartment (Fig. 2B); indicating that forced proliferations did not reduce the HSC self-renewal capacity. Of note, HSCs from the 10 × group formed a compartment that was dominated by CD150^high^ expressing cells (Fig. 2B). Unexpectedly, the reduced competitive fitness of proliferatively stressed HSCs was not accompanied by a significantly altered lineage differentiation preference (Fig. 2C), with only a statistically non-significant trend for increased myeloid bias of HSCs from the 4 × and 10 × donors. To test for the effect of replicative stress on long-term reconstitution potential we performed a secondary competitive transplant of donor-derived bone marrow. As expected for a secondary transplant of HSCs from similar aged donors as the experimental mice (middle age), we observed an overall low competitive potential for all groups but a surprisingly increased potential for the 2 × and 4 × groups (Fig. 2D).

### Modest long-term transcriptional changes after proliferative stress

**Fig. 3:**
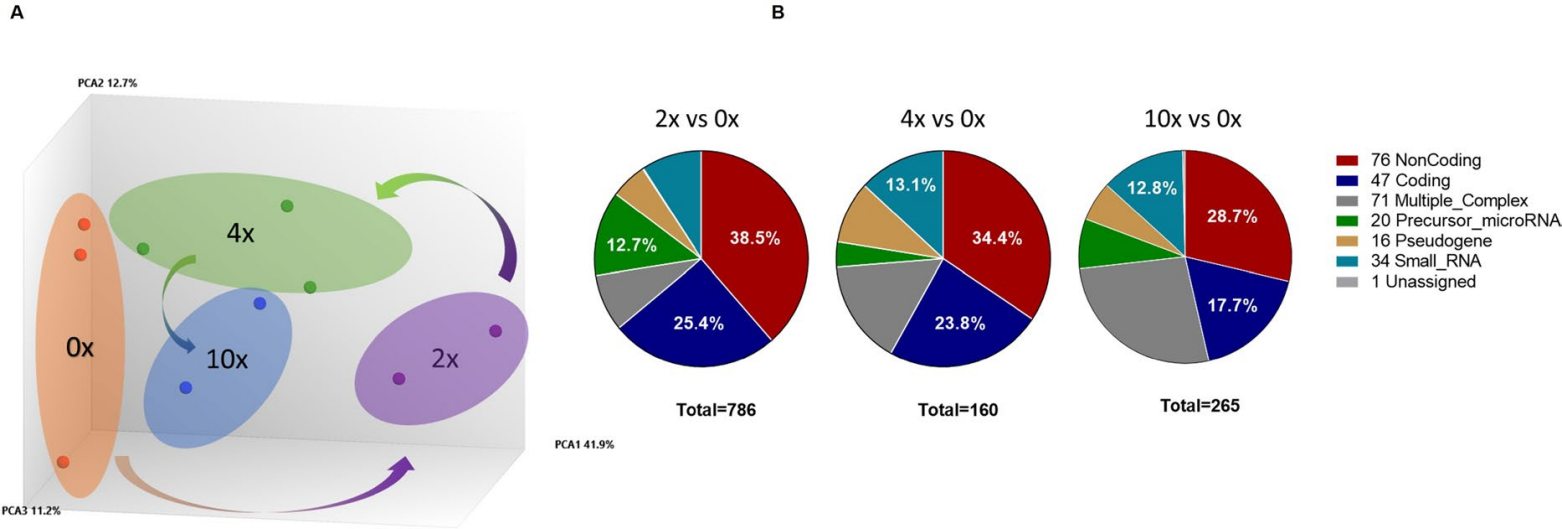
Long-term mRNA expression profile of HSCs is resilient to forced proliferations. **A** PCA plot. **B** Distribution according to the general annotation of statistically trending differentially expressed transcripts with a threshold of *p* < 0.05 without Bonferroni correction

To better understand the intrinsic changes that drive the observed functional alterations in HSCs, we examined their mRNA expression. To ensure that the analysis represents long-term effects of proliferative stress on HSCs rather than the immediate response, experiments were performed 2 months after the last 5-FU cycle. Overall, the gene expression profile correlated with experimental groups (Fig. 3A). However, differential gene expression analysis indicates gene expression changes were minimal, and often did not meet the threshold criteria for significance (Fig. S4). Similarly, no genes were detected as statistically dependent on the number of cycles in a multiple regression analysis. Even under a less stringent threshold for differential expression, the analysis was dominated by non-coding or “predicted” gene transcripts (Fig. 3B). These differentially expressed transcripts were not enriched for any annotated pathways or protein–protein interaction networks. Taken together, our results indicate a highly regulated gene expression pattern in HSCs, that is largely unperturbed following this replicative stress.

### DNA damage dynamics

**Fig. 4:**
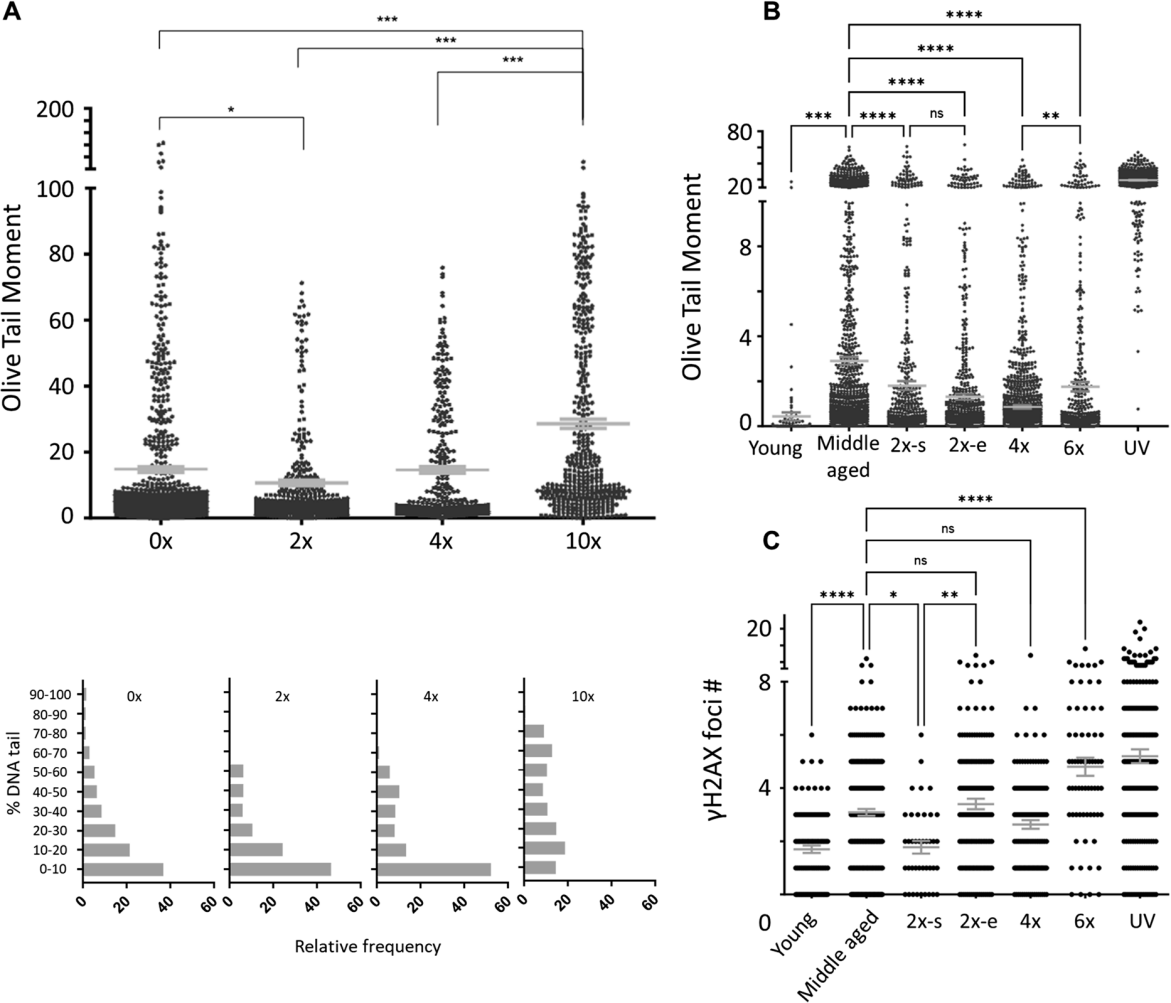
DNA damage is initially repaired upon cell proliferation but with repetitive stress begins to accumulate. **A**, **B** Olive tail moment score for an alkaline comet assay to quantify DNA damage in single HSCs, performed 1 month following treatment end. Each dot represents a single cell measured with red lines showing mean ± SEM (top panel). The bottom panel shows the %DNA in tail distribution. Results for UV positive controls in **A** are not presented due to extreme overlap in the tail but indicate a successful assay (Fig. S5A). **B** Repeat of the experiment with age controls and a treatment of 2 × at the start (2x-s) and end (2x-e) of the entire treatment regimen. **C** Quantification of γH2AX foci for the samples in **B**. Statistical analysis was performed by one-way ANOVA with multiple comparison correction

As previous studies have shown a relationship between HSC proliferation, aging, and DNA damage, we tested for genomic integrity following forced replications using the comet assay. Similarly to Beerman et al. [23], DNA damage in HSCs was reduced following forced replication (Fig. 4A, Fig. S5A). However, continuous forced proliferation resulted in a marked increase in the DNA strand break burden. This was evident in two of the most commonly reported comet parameters — Olive Tail Moment and % Tail DNA. Of note, the DNA damage profile of the 4 × group was reduced compared to the aged-matched controls, indicating that the reduced reconstitution potential described above was not mediated by genomic stress. Next, we repeated the experiment on an independent cohort with 0–6 cycles of 5FU treatments where the groups with 2 doses of 5FU are treated either in the beginning (2x-s) or end (2x-e) of the experimental period, thereby giving HSCs a long or short period of post-stimulation, respectively. In this experiment also, DNA damage that was accrued with age was repaired upon initial forced replication, a trend which was reversed by cycle 6 (Fig. 4B). It is important to note that the tail moment values are highly dependent on the experimental conditions, which resulted in differences between the experiments but in a consistent relationship to both positive and negative controls.

Interestingly, we observed a different DNA damage distribution in HSCs from mice that received the 5FU treatment in the beginning (2x-s) than the ones that were treated at the end (2x-e), suggesting DNA damage is repaired when challenged to cycle and is then accumulated again (2x-s had time to accumulate again while the other cycles are administered whereas 2x-e has just repaired the accumulated damage before quantification). These results are further reinforced when compared to the number of γH2AX foci in the HSC nuclei (Fig. 4C, Fig S5C). Overall, the results support the hypothesis that DNA damage accrues in HSCs with age and can be repaired upon cell proliferation; however, if the replication stress is excessive, DNA damage accumulates.

### Epigenetic imprint of proliferative stress

**Fig. 5:**
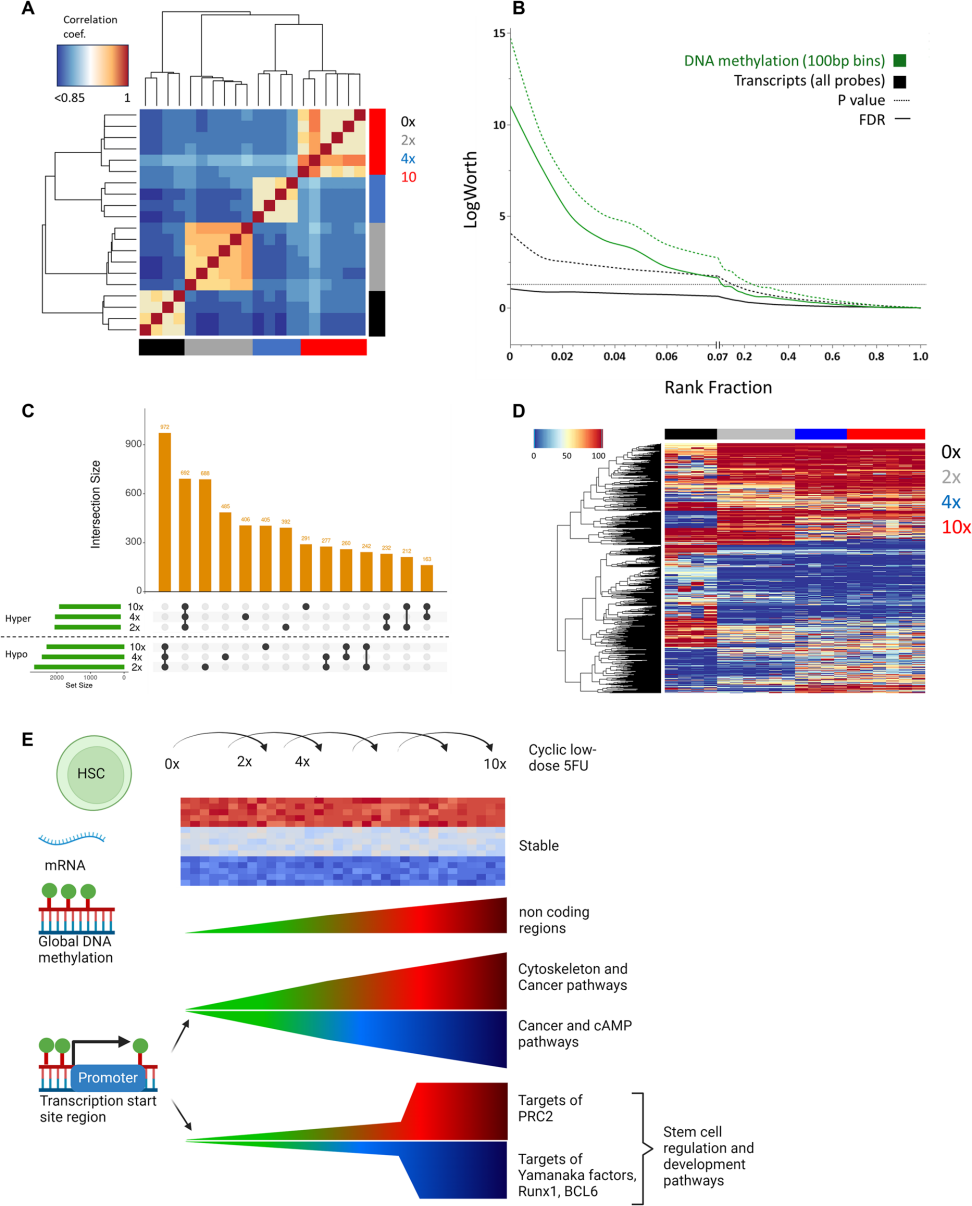
Proliferative stress is imprinted in the DNA methylome**.** HSCs sorted 1 month following end of treatment were assessed for DNA methylation by reduced representation bisulfite sequencing (RRBS). **A** Multiple correlation plot for overall DNA methylation between each sample expressed as a clustered heatmap of correlation coefficient. **B** Response screening assay to determine genes and genomic regions (100 bp wide) that change in expression or methylation levels (respectively) as a function of the number of 5FU treatment cycles. For visualization, all results were ranked by statistical significance (Rank fraction) and are presented as logworth (-log10(p) and -log10(FDR)) as a function of the rank fraction. **C** Upset plot representing the overlaps between differentially methylated regions (DMRs) in different groups. **D** Heatmap of methylation levels in DMRS that correspond to the overlaps between the comparisons of 2x, 4x, and 10 × to 0x. The color on top represents the sample group (0x = black, 2x = gray, 4x = blue, 10x = red). **E** Graphical summary of study results

As the intrinsic, reduced functional potential of the stem cells was not mediated at the mRNA expression level, and was not fully correlated with DNA integrity, we next looked at epigenetic regulation by measuring DNA methylation with reduced representation bisulfite sequencing (RRBS). Overall, DNA methylation profiles were highly dependent on the experimental group and consistent between the samples (Fig. 5A). In terms of global methylation changes, we observed a small but persistent hypermethylation correlating with proliferative stress (Fig. S6A), concordant with previous observations on HSC aging [3, 4, 35]. Interestingly, the global methylation trajectories were diverse across genomic locations, with an increase that occurred quickly after forced replication (2x) in non-coding regions, but more linearly in most other genomic regions, and promoter regions showed no global increase (Fig. S6C). Unlike mRNA expression, methylation at many DNA regions were found to be significantly dependent on the number of 5FU cycles (Fig. 5B). These cycle-dependent methylation changes were heavily concentrated around transcription start sites (TSS) (Fig. S7A, B) and were both hyper- and hypo-methylated (Fig. S7C), suggesting a coordinated response of differential methylation around promoter sites rather than a stochastic response. When performing a group comparison to quantify changes compared to untreated controls, differential methylation was again concentrated around transcription start sites, regardless of cycle number (Fig. S7D). Therefore, we further analyzed the genes whose regulation could be affected by differential methylation in their promoter region. The strongest reaction to proliferation was a common response independent of cycle number (Fig. 5C). In broad terms, these changes contained both hyper- and hypo-methylation and occurred in genes involved in cancer-related, cell cycle regulation, cytoskeleton regulation, and the cAMP pathway (Fig. S8A). Specifically, we observed a strong hypermethylation in STAT5A, IL3, CEBPE, FLT3, EIF4EBP1, RARA, GRB2, BCL2A1D, and BCL2A1B; all of which are involved in hematopoiesis regulation and leukemia in humans. Hypomethylation was specifically observed in genes critical for cell cycle regulation, especially in the context of cancer such as CDKN1B, CDKN2A, NOS2, ITGA3, PPARG, and 4 members of the WNT family (WNT5A, WNT7A, WNT1, and WNT3A). Interestingly, the promoter region of both TERT and TERC telomerase components were hypomethylated following proliferative stress.

While these methylation changes were associated with a general proliferative stress, they were not associated with the significantly reduced reconstitution potential that we detected after 4 cycles of forced proliferations in a transplant setting. Therefore, we looked for differentially methylated genes that were unique to the 4 × and 10 × groups compared to the baseline (0x). These 214 hypermethylated and 163 hypomethylated genes (Fig. 5C, D) were highly enriched for processes relating to stem cell differentiation and surprisingly, developmental processes (Fig. S8B). Notably, analysis of the regulatory elements that bind the differentially methylated regions with ChEA (Fig. S8C) shows a strong enrichment for EZH2 and SUZ12; both of which are components of the polycomb complex PRC2 [40]. Gene promoter regions that were hypomethylated in both 4 × and 10 × groups, but not in 2x, were especially interesting as they were highly enriched for targets of 3 Yamanaka factors (*Klf4*, *Nanog*, *Oct4*) that are not normally active in HSCs. We also noted an enrichment in targets of RUNX1 and BCL6. Interestingly, the latter coincides with an exceptionally strong hypermethylation around the promoter region of *Bcor*, a co-repressor that is highly specific in binding to BCL6 [41] (Fig. S8D).

Overall, we find that DNA methylation was highly responsive to forced replications in HSCs, both in general terms and in a manner that correlates to observed functional changes, suggesting that epigenetic regulation is the long-term mediator of the proliferative stress memory in blood stem cells (Fig. 5E).

## Discussion

Our first goal in this study was to examine whether aging of HSCs is, or can be, driven by proliferative stress. We found that forcing HSCs to proliferate in vivo mimics some, but not all, aging phenotypes, in a cycle-dependent manner. For example, while it seems forced proliferations resulted in reduced ability of HSCs to differentiate into the lymphoid lineage (as evident in the frequencies of Flk2 + MPPs, CLPs, and peripheral blood lymphocytes), we did not observe a marked increase of myeloid cell composition in the blood. A possible explanation for this is that some aging phenotypes occur relatively early in a manner that superseded the effects of proliferative stress, and since the duration of these experiments caused these animals to be near middle age at time of analysis, aging may be the dominant feature. Alternatively, it is likely that proliferation of HSCs accounts for a portion, but not the whole of aging phenotypes. It is also important to note that we observed aging phenotypes that appeared relatively early in our mice (early middle age) and were further exacerbated by forced HSC proliferation. Most notable of these was the accumulation of CD150^high^ HSCs, a phenotype that appears to be intrinsic to the stem cells as it was retained in a transplant setting. Accordingly, we find the proliferatively stressed HSCs are potent in their ability to self-renew (as evident by a high HSC chimerism in a transplant) but suffer from a deficit in their ability to differentiate and reconstitute the rest of the blood system—both features of physiologically aged HSCs.

Our second goal was to better understand the dynamics of genomic integrity following proliferation in aging HSCs, especially due to the previous inconsistencies in observations between different groups. By creating a differential proliferative load using cyclic low dose-5FU treatments, we affirm that DNA damage (as detected by comet assay) is accrued in aging HSCs; that damage is mostly repaired upon initial proliferation but begins to accumulate with severe proliferative stress. Our results also indicate that DNA damage, as measured by strand breaks, is not the main driver of the reduced functional potential of exhausted HSCs. This was evident in the fact the HSCs from the 4 × group had comparable damage to controls but a markedly reduced potential in a transplant. It is important to note however that repair of the age-accrued damage during DNA replication can result in a variety of DNA mutations, which could explain a reduced potential in the 4x, independent of the damage assayed for in the comet assay. However, such mutations could also result in a selective advantage, as observed in clonal hematopoiesis of indeterminate potential (ChIP) [42], and therefore mutation accumulation is likely not a major driver of the observed loss of potential.

While we did not measure multiple epigenetic regulators, we find multiple lines of evidence that DNA methylation is a candidate to mediate the proliferative “memory” of HSCs: (i) DNA methylation was highly dependent on the number of cycles of forced proliferation. (ii) DNA methylation was strongly correlated with the functional transplant assay. (iii) DNA methylation as a function of proliferative stress was highly focused around promoter regions in a manner that suggests a coordinated response rather than stochastic changes. We find it particularly interesting that HSCs in homeostasis all presented with a similar mRNA expression profile, regardless of proliferative history. This result indicates that, as previously proposed [3], the DNA methylation changes in HSCs are impacting their potential rather than directly influencing their operational mechanisms. An important note to our observations on RNA transcripts is that we did observe changes in non-coding and poorly annotated transcripts. As there is little available information regarding their function, it would be interesting to investigate more closely what these transcripts contribute to, as they could serve as potential regulators of stem cell function. The exceptionally high proportion of differentially methylated promoters in genes that are targets of the polycomb repressive complex 2 (PRC2) is especially interesting as PRC2 has been implicated in aging in general [43], stem cell aging [44], and in regulating HSC self-renewal and differentiation [45–47]. It was beyond the scope of this study to investigate the H3K27 trimethylation profile in HSCs, but given the implication of PRC2, it would be interesting to see how the histone modifications change in response to proliferative stress in the long-term.

The quantification of cell populations at homeostasis, during aging, and following HSC transplants, indicates that proliferative stress in HSCs could have negative ramifications on the immune system. This was evident in the accumulation of CD150^high^ HSCs, a reduced fraction of MPP Flk2^+^, CLPs, and circulating lymphocytes. However, we did not perform immune challenge assays and can therefore not absolutely define that impact. Regardless, this is especially important in the context of clinical stem cell transplants, where a history of massive HSC proliferation (to reconstitute the host hematopoietic system) could result in reduced immune potential. Indeed, it would be interesting to investigate whether increased cycling of human progenitor cells have similar negative consequences as this could have clinical consequences for transplantation: fewer donor HSCs would require higher proliferative stress to effectively reconstitute the hematopoietic system. If so, we would suggest that a paradigm of transplant using higher HSC number could result in a lower proliferative load and improved outcomes.

Overall, we find that HSC proliferation is initially associated with repairing age-accrued DNA damage without a decisive impact on their functional potential. However, we also find that persistent proliferative stress eventually promotes DNA damage accumulation and a reduced functional potential carried by intrinsic changes including an accumulation of CD150^high^ HSCs. This intrinsic proliferative stress “memory” is likely mediated by DNA methylation changes that involve the PRC2 complex and developmental programs.

## Supplementary Information

Below is the link to the electronic supplementary material.

Supplementary file1 (PDF 1630 KB)

## Data Availability

All data in this study is available as supplementary data or in the indicated repositories.
