# Supplementary material for: DNA methylation drives hematopoietic stem cell aging phenotypes after proliferative stress
Source: GeroScience. 2024 Oct 11;47(2):1873–86. doi: 10.1007/s11357-024-01360-4 (PMC11978565; doi:10.1007/s11357-024-01360-4)

Supplementary Fig. 1

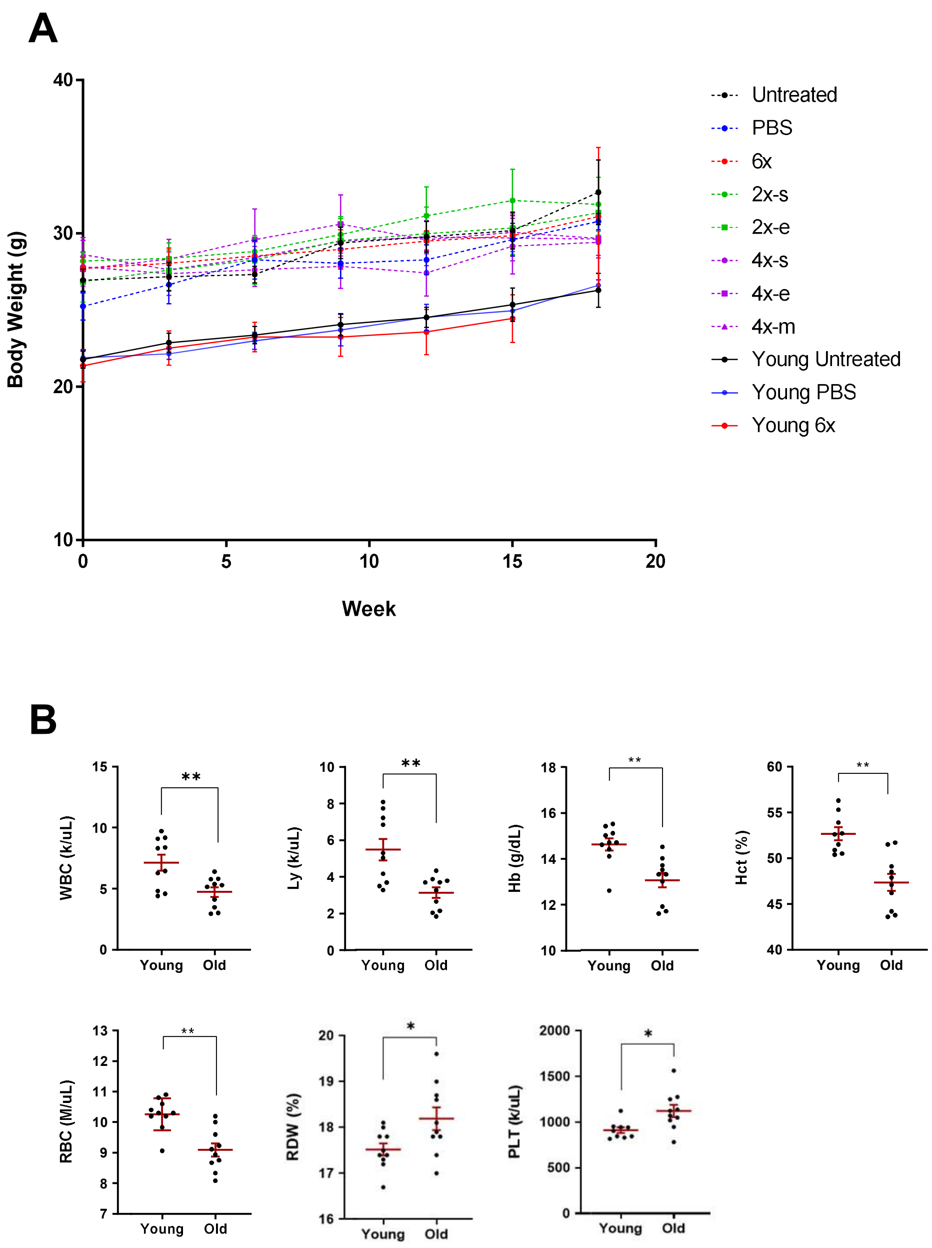

Supplementary Fig. 2

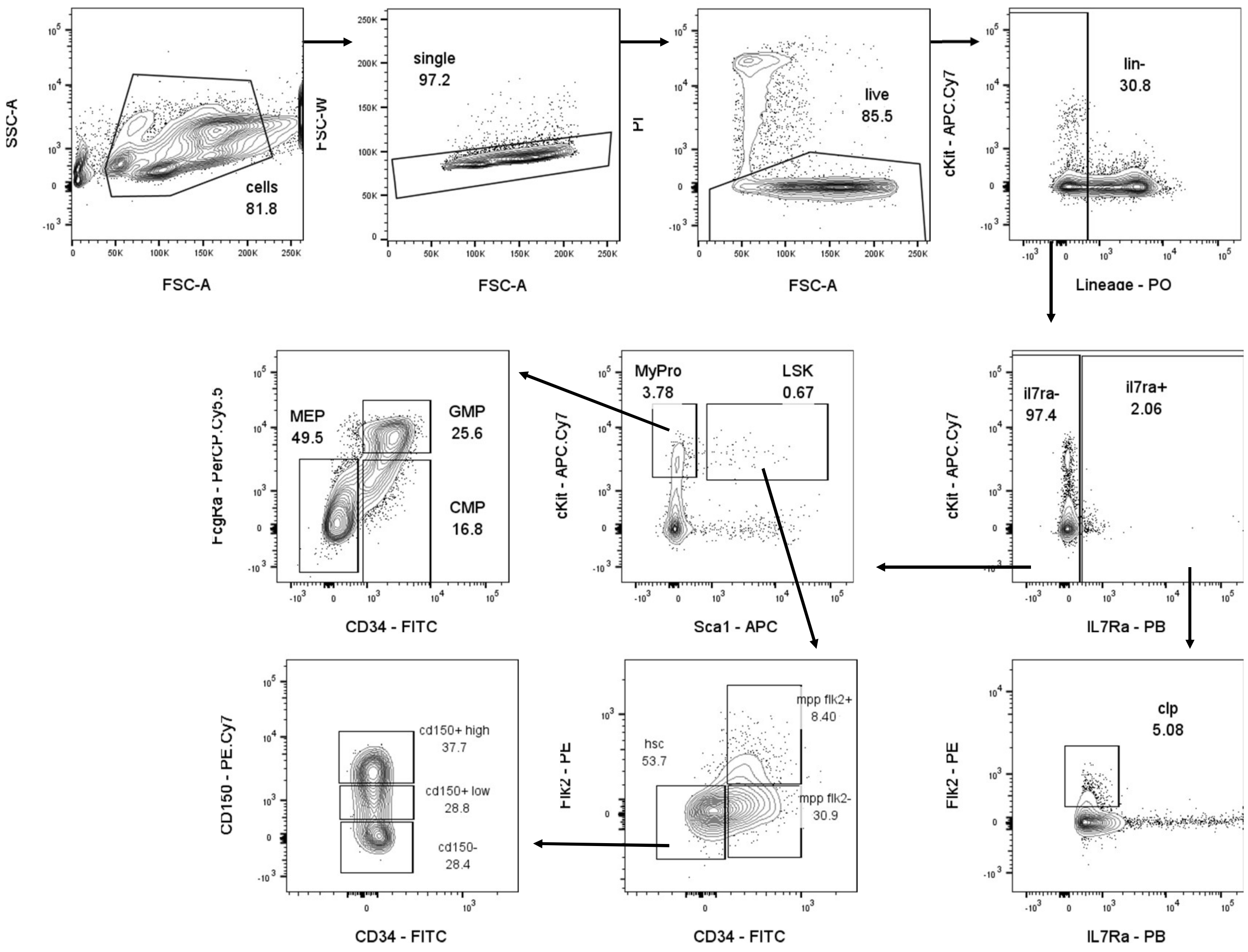

Supplementary Fig. 3

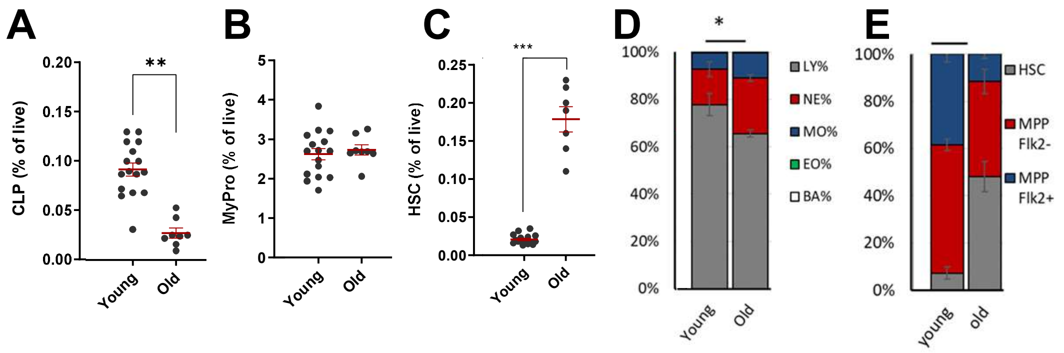

Supplementary Fig. 4

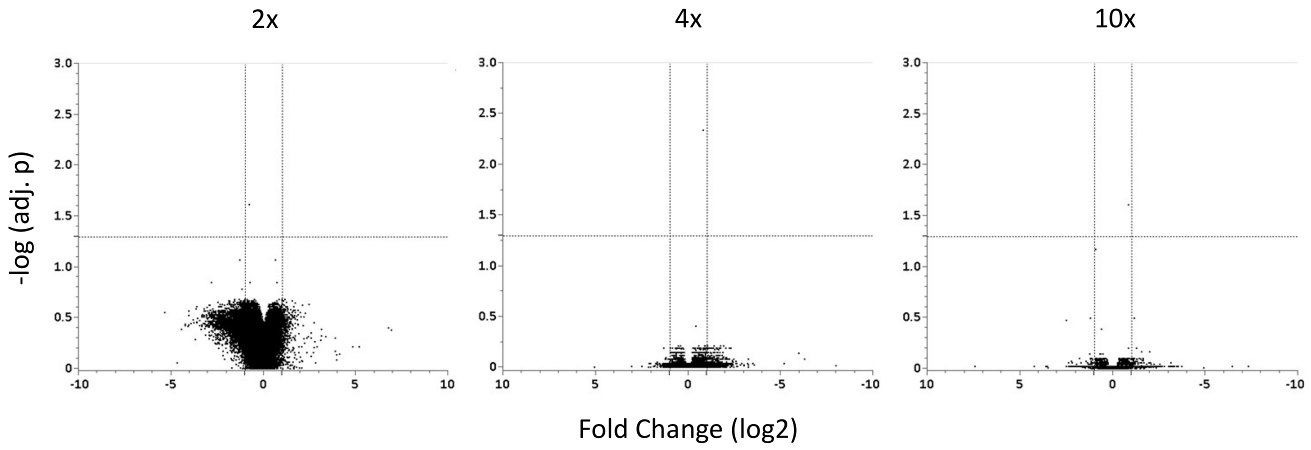

Supplementary Fig. 5

A

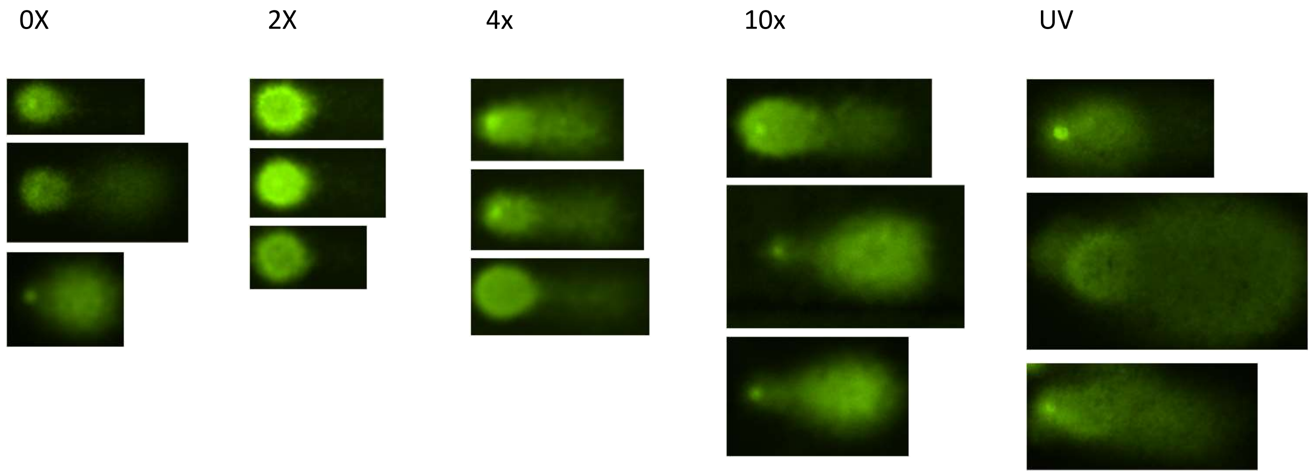

B

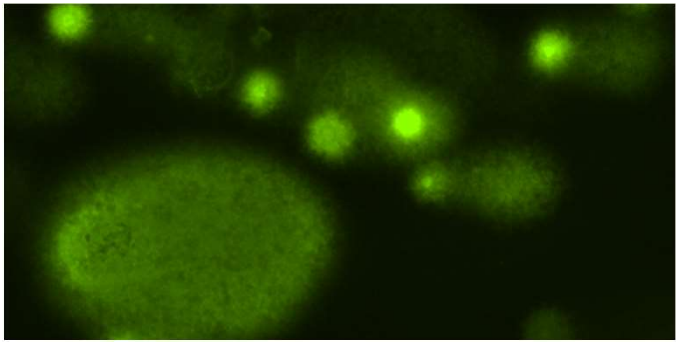

B

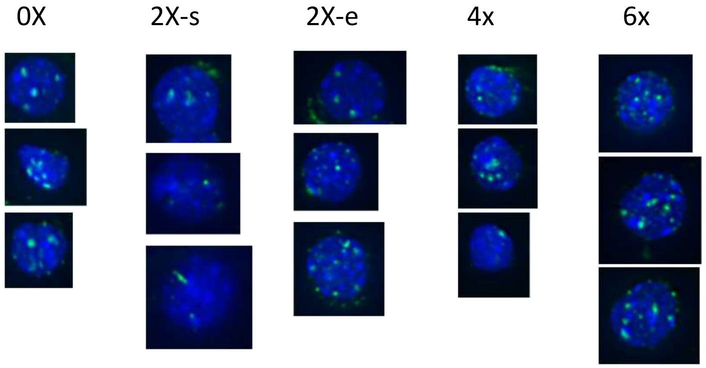

Supplementary Fig. 6

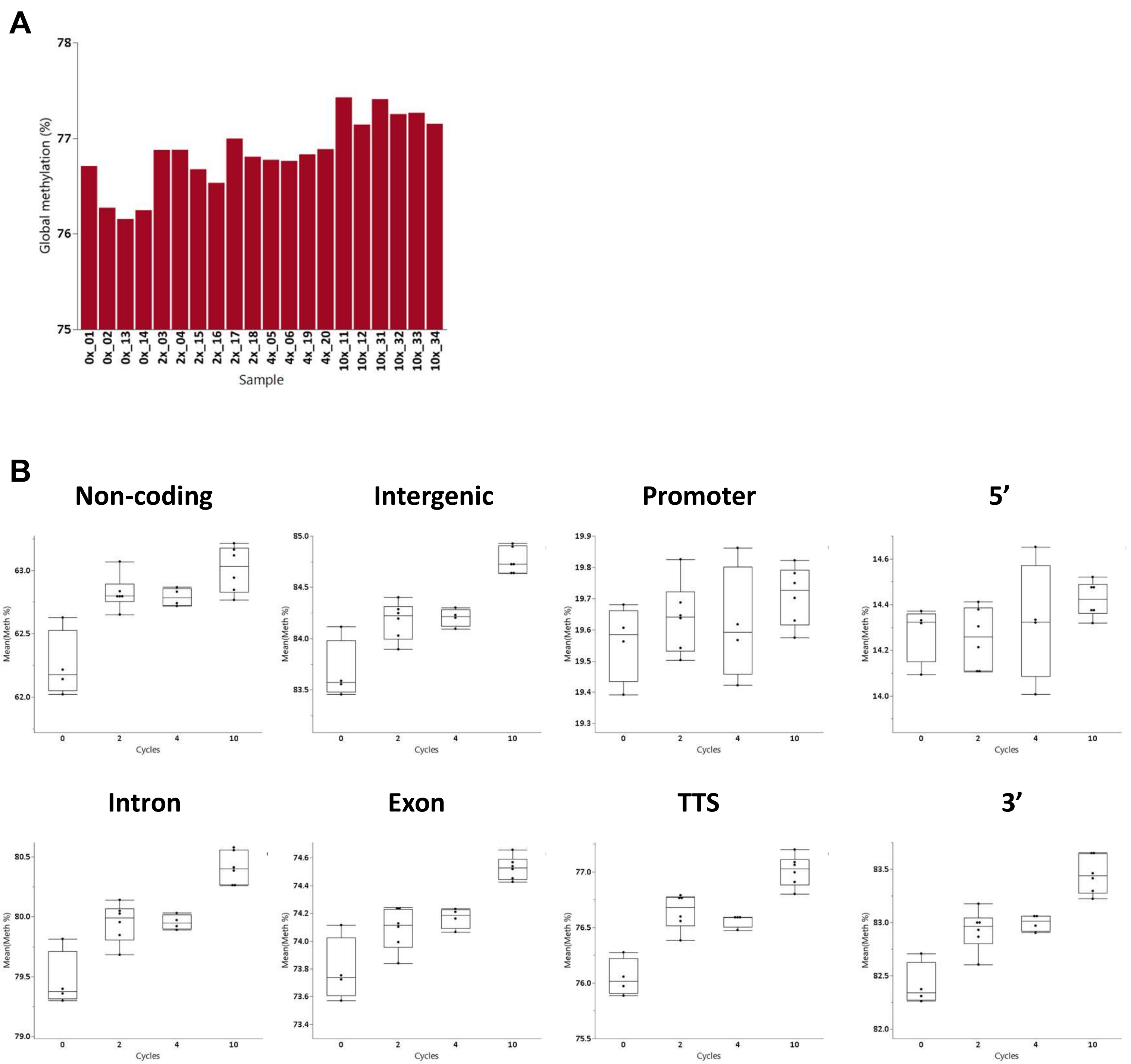

Supplementary Fig. 7

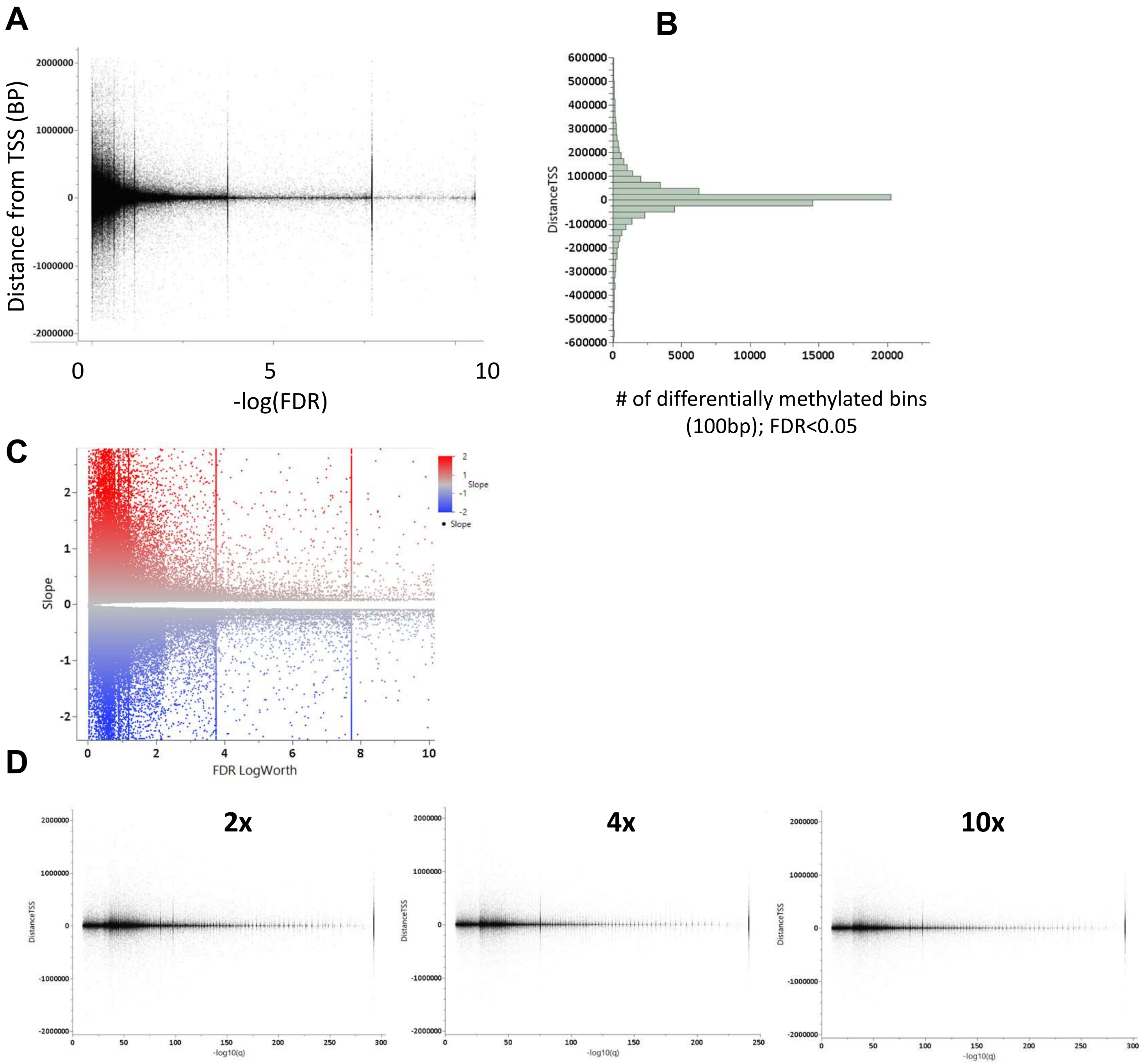

Supplementary Fig. 8

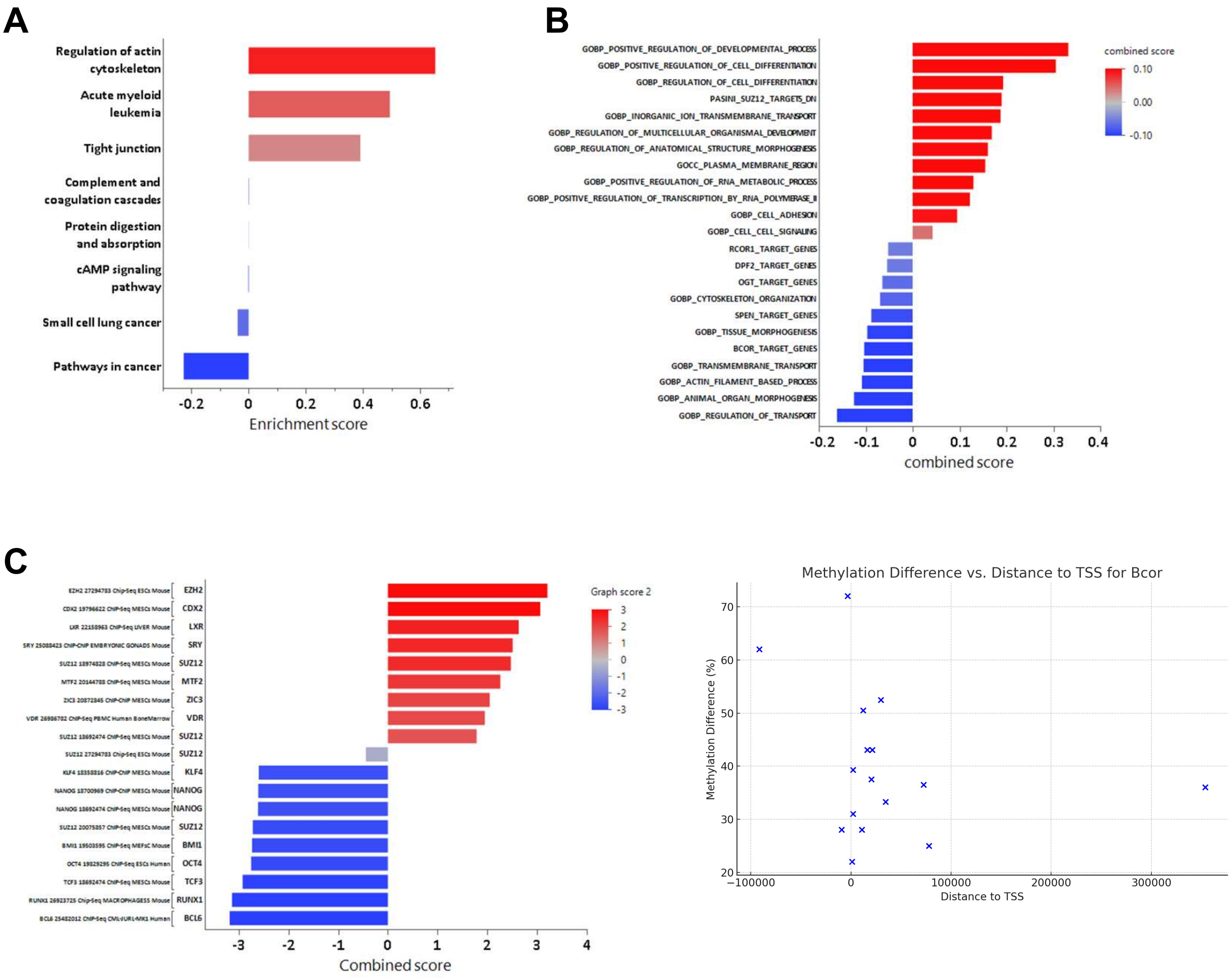

Supplement: Supplementary file 1 — Supplementary file1 (PDF 1630 KB) [file 11357_2024_1360_MOESM1_ESM.pdf]
